# Supplementary material for: Prediction of allosteric sites and signaling: Insights from benchmarking datasets
Source: Patterns (N Y). 2021 Dec 9;3(1):100408. doi: 10.1016/j.patter.2021.100408 (PMC8767309; doi:10.1016/j.patter.2021.100408)
Supplement: Document S1. Tables S1 and S5 [file mmc1.pdf]

Patterns, Volume *3*

Supplemental information

Prediction of allosteric sites and signaling: Insights from benchmarking datasets

Nan Wu, Léonie Strömich, and Sophia N. Yaliraki

Table S1: Performance of other computational methods in prediction of allosteric sites. These methods have used ASBench[1] and AlloSteric Database (ASD)[2] for method validation.

Methods	Prediction Accuracy	Remarks
PARS[3]	65%	The method was tested on 58 proteins collected from the ASD[2].
AlloPred[4]	59%	119 proteins were collected from ASBench[1] and relevant site information were retrieved from UniProt[5] and the Catalytic Site Atlas[6]. Only the chain(s) involving the orthosteric and allosteric sites and the connecting chain(s) were considered i.e. not the whole protein structure. An average prediction accuracy of 59% was achieved when tested on 40 proteins (79 was used for model training).
AllositePro[7]	51.7%	The 147 nonredundant allosteric sites from the Core-Diversity set of ASBench[1] were used in this study and 76 out of 147 allosteric sites was successfully predicted.
SBSMMA[8]	Not reported	41 proteins were selected based on the operational definition of allosteric sites in the paper from ASBench[1]. Predictive power is quantifiled by the area under the ROC curves (AUCs) and 28 out of 48 have an AUC above 0.6.

Table S5: Protein structure used from the CASBench database. Orthosteric and allosteric ligands and site residues can be found directly in the CASBench database.

cas number	PDB
**cas0001**	1nxe, 1nxg, 1owc, 4g6b, 4jad, 4jae, 4jaf, 4jag
**cas0002**	3i1y, 3i28, 3koo, 3otq, 5ahx, 5ai4, 5ai5, 5aia, 5ak4, 5ak5, 5ake, 5akh, 5akx, 5aky, 5ald, 5alf, 5alh, 5alm, 5aln, 5alo, 5alt, 5alu, 5alv, 5alw, 5aly, 5am0, 5am4, 5am5
**cas0003**	3ion, 3iop, 3rwp, 4rqk, 4rqv, 4rrv, 4xx9
**cas0004**	4ey5, 5hf6, 5hf8, 5hf9, 5hfa
**cas0010**	1i2d
**cas0011**	2ewn
**cas0015**	2ym4, 2ym8, 4fst, 4fsy, 4ft3, 4ft7, 4fta, 4ftn, 4fto, 4ftr, 4ftu, 4gh2
**cas0016**	3csm, 4csm
**cas0021**	3l9h, 4zhi
**cas0024**	4ald
**cas0027**	1cza, 1dgk
**cas0028**	1lld, 1lth
**cas0029**	1ldn
**cas0030**	2him, 2p2d
**cas0039**	3ddn
**cas0040**	1psd, 1yba, 2p9c, 2p9e, 2p9g, 2pa3
**cas0047**	4r1r
**cas0050**	1xtu, 1xtv
**cas0051**	11ba, 11bg, 1bsr, 1n3z, 1r3m, 1r5c, 1tq9, 3bcm, 3djo, 3djp, 3djq, 3djv, 3djx, 4n4c
**cas0052**	2z60
**cas0054**	2jfx, 2jfy, 2jfz, 4b1f
**cas0056**	3hmi

**cas0060**	1boz, 1dhf, 1dlr, 1dls, 1drf, 1hfp, 1hfq, 1hfr, 1kms, 1mvs, 1mvt, 1ohj, 1ohk, 1pd8, 1pd9, 1s3u, 1s3v, 1s3w, 1u71, 1u72, 2c2s, 2c2t, 2dhf, 2w3a, 2w3m, 3f8y, 3f8z, 3fs6, 3ghc, 3ghw, 3gi2, 3gyf, 3l3r, 3n0h, 3ntz, 3nu0, 3nxr, 3nxt, 3nxv, 3nxx, 3nxy, 3nzd, 3oaf, 3s3v, 3s7a, 4ddr, 4g95, 4kd7, 4keb, 4m6k, 4m6l, 4qjc, 5hpb, 5hqy, 5hqz, 5hsr, 5hsu, 5ht4, 5ht5, 5hui, 5hvb, 5hve
**cas0061**	1dre, 1ra2, 1ra3, 1ra8, 1rb2, 1rb3, 1rc4, 1rd7, 1re7, 1rg7, 1rh3, 1rx4, 1rx5, 1rx6, 1rx7, 3dau, 4ej1, 4fhb, 4i13, 4i1n, 4kjj, 4kjl, 4p3r, 4qle, 4qlg, 4x5f, 4x5g, 4x5h, 4x5i, 4x5j, 5cc9, 5ccc, 7dfr
**cas0067**	2psq, 3ril
**cas0070**	1ibc, 1ice, 1rwm, 1rwn, 1rwo, 1rwv, 2h4w, 2h4y, 2h51, 2h54, 2hbq, 2hbr, 2hby, 2hbz, 3d6f, 3d6h, 3d6m
**cas0071**	1i4o
**cas0074**	1ibv, 1ibw, 1pya
**cas0079**	1bzc, 1bzj, 1c83, 1c84, 1c85, 1c86, 1c87, 1c88, 1ecv, 1g1g, 1g7g, 1gfy, 1kak, 1kav, 1l8g, 1nwe, 1ptt, 1ptu, 1ptv, 1q1m, 1xbo, 2azr, 2b07, 2bge, 2cm7, 2cma, 2h4g, 2h4k, 2hb1, 2nt7, 2nta, 2qbp, 2qbq, 2qbr, 2qbs, 2veu, 2vey, 2zn7, 4i8n
**cas0080**	1i00, 1juj, 3ob7, 5hs3, 5x5q, 5x67
**cas0085**	1fuo, 1fup, 1fuq, 1kq7
**cas0086**	1gos, 1oj9, 1oja, 1ojc, 1s2q, 1s2y, 1s3b, 1s3e, 2bk3, 2bk4, 2bk5, 2byb, 2c64, 2c65, 2c66, 2c67, 2c70, 2c72, 2c73, 2c75, 2c76, 2v5z, 2v60, 2v61, 2vrl, 2vrm, 2vz2, 2xcg, 2xfn, 2xfo, 2xfp, 2xfq, 2xfu, 3po7, 3zyx, 4a79, 4a7a, 4crt, 5mrl
**cas0091**	1fin, 2b54, 2cch, 4ez7

References

1. Huang, W., G. Wang, Q. Shen, X. Liu, S. Lu, L. Geng, Z. Huang, and J. Zhang (Mar. 2015). "AS-Bench: benchmarking sets for allosteric discovery". *Bioinformatics* 31.15, pp. 2598–2600. DOI: 10.1093/bioinformatics/btv169.
2. Huang, Z., L. Zhu, Y. Cao, G. Wu, X. Liu, Y. Chen, Q. Wang, T. Shi, Y. Zhao, Y. Wang, W. Li, Y. Li, H. Chen, G. Chen, and J. Zhang (Nov. 2010). "ASD: a comprehensive database of allosteric proteins and modulators". *Nucleic Acids Research* 39.suppl_1, pp. D663–D669. DOI: 10.1093/nar/gkq1022.
3. Panjkovich, A. and X. Daura (2012). "Exploiting protein flexibility to predict the location of allosteric sites". *BMC Bioinformatics* 13.1, p. 273. DOI: 10.1186/1471-2105-13-273.
4. Greener, J. G. and M. J. E. Sternberg (2015). "AlloPred: prediction of allosteric pockets on proteins using normal mode perturbation analysis". *BMC Bioinformatics* 16.1, p. 335. DOI: 10.1186/s12859-015-0771-1.
5. Consortium, T. U. (Jan. 2015). "UniProt: a hub for protein information". *Nucleic Acids Research* 43.D1, pp. D204–D212. DOI: 10.1093/nar/gku989.
6. Furnham, N., G. L. Holliday, T. A. P. de Beer, J. O. B. Jacobsen, W. R. Pearson, and J. M. Thornton (Jan. 2014). "The Catalytic Site Atlas 2.0: cataloging catalytic sites and residues identified in enzymes". *Nucleic Acids Research* 42.D1, pp. D485–D489. DOI: 10.1093/nar/gkt1243.
7. Song, K., X. Liu, W. Huang, S. Lu, Q. Shen, L. Zhang, and J. Zhang (Sept. 2017). "Improved Method for the Identification and Validation of Allosteric Sites". *Journal of Chemical Information and Modeling* 57.9, pp. 2358–2363. DOI: 10.1021/acs.jcim.7b00014.
8. Tee, W.-V., E. Guarnera, and I. N. Berezovsky (June 2018). "Reversing allosteric communication: From detecting allosteric sites to inducing and tuning targeted allosteric response". *PLOS Computational Biology* 14.6, e1006228. DOI: 10.1371/journal.pcbi.1006228.
