# Supplementary material for: Prediction of allosteric sites and signaling: Insights from benchmarking datasets
Source: Patterns (N Y). 2021 Dec 9;3(1):100408. doi: 10.1016/j.patter.2021.100408 (PMC8767309; doi:10.1016/j.patter.2021.100408)
Supplement: Document S2. Article plus supplemental information [file mmc7.pdf]

Patterns

Article

Prediction of allosteric sites and signaling: Insights from benchmarking datasets

Graphical Abstract

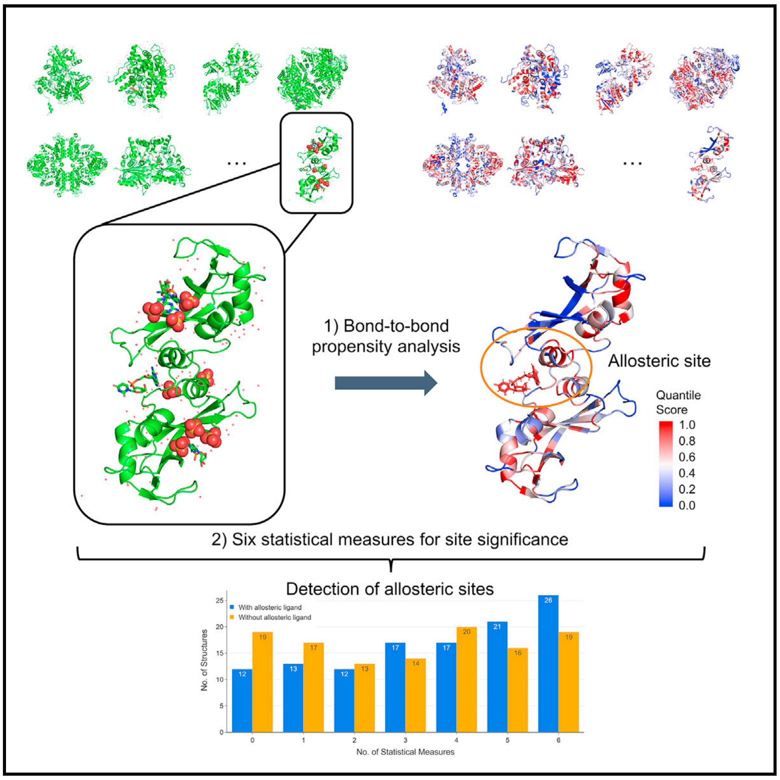

Highlights

- State-of-the-art prediction accuracy of allosteric sites on benchmarking datasets
- Multiple measures to capture different mechanistic insights in allostery
- Provide guidance for molecular design using key interaction residues

Authors

Nan Wu, Léonie Strömich, Sophia N. Yaliraki

Correspondence

s.yaliraki@imperial.ac.uk

In brief

Modulation of protein activity is important for disease treatment, and challenges associated with common drugs have fueled the need for allosteric drugs that bind distant from the active site. Yet, allosteric site prediction remains challenging. In this study, we benchmark an atomistic, graph-theoretical method called bond-to-bond propensity against allosteric protein databases to illustrate its capability to accurately uncover allosteric sites. Our six scoring measures provide additional insights into potential mechanisms involved and could guide the design of drug molecules.

Wu et al., 2022, Patterns *3*, 100408
January 14, 2022 © 2021 The Authors.
https://doi.org/10.1016/j.patter.2021.100408

CellPress

Patterns

CellPress
OPEN ACCESS

Article

Prediction of allosteric sites and signaling: Insights from benchmarking datasets

Nan Wu,[1] Léonie Strömich,[1] and Sophia N. Yaliraki[1,2,*]
[1]Department of Chemistry, Imperial College London, London W12 0BZ, UK
[2]Lead contact
*Correspondence: s.yaliraki@imperial.ac.uk
https://doi.org/10.1016/j.patter.2021.100408

THE BIGGER PICTURE Proteins are the most common drug targets, and allostery plays a key role in regulating protein activities. Predicting allosteric sites *in silico* is of great interest in expanding the chemical space of drug discovery. Here, we demonstrate how bond-to-bond propensity analysis is used to not only predict allosteric sites in large benchmarking datasets but also to shed light on the possible allosteric mechanisms involved. Our quantitative analysis of a given site with a range of statistical measures allows the identification of key residues required for allosteric signaling. The data can be harnessed for artificial-intelligence-driven drug discovery and digital molecular design, which are new areas of interest in the data science community.

1 **2** 3 4 5 **Proof-of-Concept:** Data science output has been formulated, implemented, and tested for one domain/problem

SUMMARY

Allostery is a pervasive mechanism that regulates protein activity through ligand binding at a site different from the orthosteric site. The universality of allosteric regulation complemented by the benefits of highly specific and potentially non-toxic allosteric drugs makes uncovering allosteric sites invaluable. However, there are few computational methods to effectively predict them. Bond-to-bond propensity analysis has successfully predicted allosteric sites in 19 of 20 cases using an energy-weighted atomistic graph. We here extended the analysis onto 432 structures of 146 proteins from two benchmarking datasets for allosteric proteins: ASBench and CASBench. We further introduced two statistical measures to account for the cumulative effect of high-propensity residues and the crucial residues in a given site. The allosteric site is recovered for 127 of 146 proteins (407 of 432 structures) knowing only the orthosteric sites or ligands. The quantitative analysis using a range of statistical measures enables better characterization of potential allosteric sites and mechanisms involved.

INTRODUCTION

Proteins are ubiquitous in all aspects of cellular life where they fulfil crucial functions, while their malfunction could result in disease states.[1,2] By 2017, 70% of small molecule drugs on the market targeted four types of proteins, namely protein kinases, ion channels, rhodopsin-like G protein-coupled receptors, and nuclear hormone receptors.[3] Most current small molecule drugs modify or inhibit the action of a protein by directly binding to the primary active site (also known as the orthosteric site) of the protein. The main advantage of this drug type is the high affinity and generally high specificity toward the orthosteric site as proved by a large number of successful drugs on the market.[4] Despite such advantages, the configuration of orthosteric sites is similar for proteins performing related functions, and a low selectivity leads to off-target toxicity.[5] For instance, orthosteric sites for adenosine triphosphate binding in different kinases are similar, making the optimization of selective kinase inhibitors challenging.[6] In addition, prolonged exposure to the drugs results in drug resistance, through either modifications of the drug molecules[7] or changes to the orthosteric sites.[8–12] Moreover, orthosteric drugs act as complete inhibitors or activators rather than modulators of proteins, so their therapeutic effect may not be the most optimal.[10]

Allostery broadly refers to the modulation of protein activity when achieved through binding at a distinct site from the

This is an open access article under the CC BY license (http://creativecommons.org/licenses/by/4.0/).

orthosteric site.[13] These binding events may result in conformational changes of the targeted proteins and affect the binding of natural substrates to orthosteric sites. Conformational modification can enhance or reduce the binding affinity of natural substrates at orthosteric sites and can, therefore, lead to a controlled upregulation and downregulation of protein activities, which is difficult to achieve by orthosteric site binding.[14] Allosteric modulators therefore have a lower potential for adverse side effects. Once all the allosteric sites are fully occupied, the drug reaches saturation (a ceiling level), and there is no further pharmacological effect. This indicates that on-target safety can be guaranteed.[15,16] Contributing to the low off-target effects of allosteric drugs is the low evolutionary pressure for allosteric sites to accommodate an endogenous substrate compared with the well-conserved orthosteric sites.[17] This would allow for highly selective drug targeting in closely related protein families by exploiting allosterism. Despite some chemical and pharmacological issues associated with allosteric regulators including intractable structure-activity relationships and ligand-biased signaling,[18] allosteric modulators still provide significant benefits over orthosteric regulators.

The two main challenges for using allostery in drug development are finding suitable allosteric sites in the first place and designing molecules that bind and exert modulation effects. The design of allosteric site binders could follow well-established approaches used to develop molecules that bind to orthosteric sites, such as high-throughput screening,[19] structure-based drug design,[20] and peptide phage display.[21] To achieve a high specificity as well as the intended modulation, it is indispensable to search for unique allosteric sites for the targeted protein. Therefore, efficient and effective methods for identifying putative allosteric sites are of great interest to guide the rational design of allosteric modulators and contribute to the field of drug discovery and development.[22]

Experimental methods including tethering,[23,24] nuclear magnetic resonance,[25,26] and high-throughput screening followed by X-ray crystallography[27,28] have successfully led to the discovery of a few novel allosteric sites. However, these methods involve screening of large compound libraries, which is laborious and time-consuming. To circumvent the challenges associated with the experimental methods, numerous computational methods have been developed to predict allosteric sites (reviewed in Collier and Ortiz[29] and Sheik et al.[30]) with various degrees of success. The continuous growth of the Allosteric Database (ASD), which contains data of 1,949 allosteric proteins, their binding sites, and other relevant information,[31–33] and the construction of benchmarking datasets for allosteric proteins, ASBench[34] and CASBench,[35] have provided comprehensive resources in aiding the identification of allosteric sites with computational methods.

There are two general ways of approaching the problem of identifying putative allosteric sites computationally: (1) identifying allosteric sites without considering the communication with orthosteric sites and (2) uncovering the allosteric communication pathways between orthosteric and allosteric sites.[36] Several studies have followed the first approach; Huang et al. developed Allosite to find allosteric sites based on topological and physicochemical characteristics of allosteric and non-allosteric sites using a support vector machine classifier,[37] while Chen et al. built a random forest model that utilized calculated descriptors of orthosteric, allosteric, and regular sites (binding sites without any function) and their bound ligands to classify potential sites on a given protein and identify putative allosteric sites.[38] Similarly, not concentrating on cognate ligands, Fogha et al. performed computational analysis of the density and clustering of crystallization additives that are used to stabilize proteins during the process of crystallization.[39] These methods, although achieving some promising predictability for putative allosteric sites, focus merely on the potential binding pockets on the protein and do not consider the effects of binding at these sites on the protein, which is the key concept of allostery. Therefore, these approaches alone are not sufficient to identify potential allosteric sites. Molecular dynamics (MD) simulations and normal mode analysis (NMA) of elastic network models (ENM) are widely used within the second approach of identifying allosteric signaling paths based on protein dynamics described by Newton's equation of motion. MD simulations can be applied to model proteins at atomic resolution and aid the understanding of communication pathways in proteins.[40,41] For example, Shukla et al. applied MD simulations to reveal the structures of intermediates of a non-receptor tyrosine kinase c-Src and analyzed its activation pathways to discover inhibitory allosteric sites.[42] However, MD simulations require a vast amount of computational resources if applied at an atomistic level for large proteins, and applying conventional all-atom MD simulations to access the timescales of ligand-binding processes of proteins would not be computationally feasible.[43] To retain crucial characteristics of dynamics but also alleviate high computational demands, ENM was introduced.[44] Performing NMA of ENM on proteins provides access to global modes of the structures and results in good agreement on large-scale motions with MD simulations.[45–47] Most available methods include NMA of ENM as the main component and use a perturbation approach to measure the response of the protein to ligand binding or unbinding,[36] thereby predicting allosteric sites, such as PARS.[48,49] The results obtained from NMA of ENM can be combined with machine learning for the identification of allosteric sites and have been applied in AlloPred[50] and AllositePro.[51] Guarnera and Berezovsky introduced a structure-based statistical mechanical model of allostery (SBSMMA) that differs from ENM[52] to predict allosteric sites[53] through the calculation of allosteric potential.[54,55] Although both ENM and SBSMMA are successful in modeling proteins and require much less computational power than MD simulations, they have two inherent limitations: not providing atomistic details of the protein and not considering long-range interactions greater than a certain distance. A key limitation associated with both ENM and SBAMMA is the presence of cutoff distances for the harmonic interactions as the proteins represented by these two models are coarse grained at the residue level. ENM treats each residue as a mass and represents a protein as a network of masses connected by virtual strings if they are within a cutoff distance.[56] SBSMMA uses the coarse-grained representation of proteins based on $C\alpha$ harmonic models, and residues in contact must have their $C\alpha$ atoms within a cutoff distance of 11 Å.[52] As a result subtle changes in protein conformations cannot be captured.

Bond-to-bond propensity analysis was introduced recently to circumvent these limitations, mainly to retain atomistic detail and

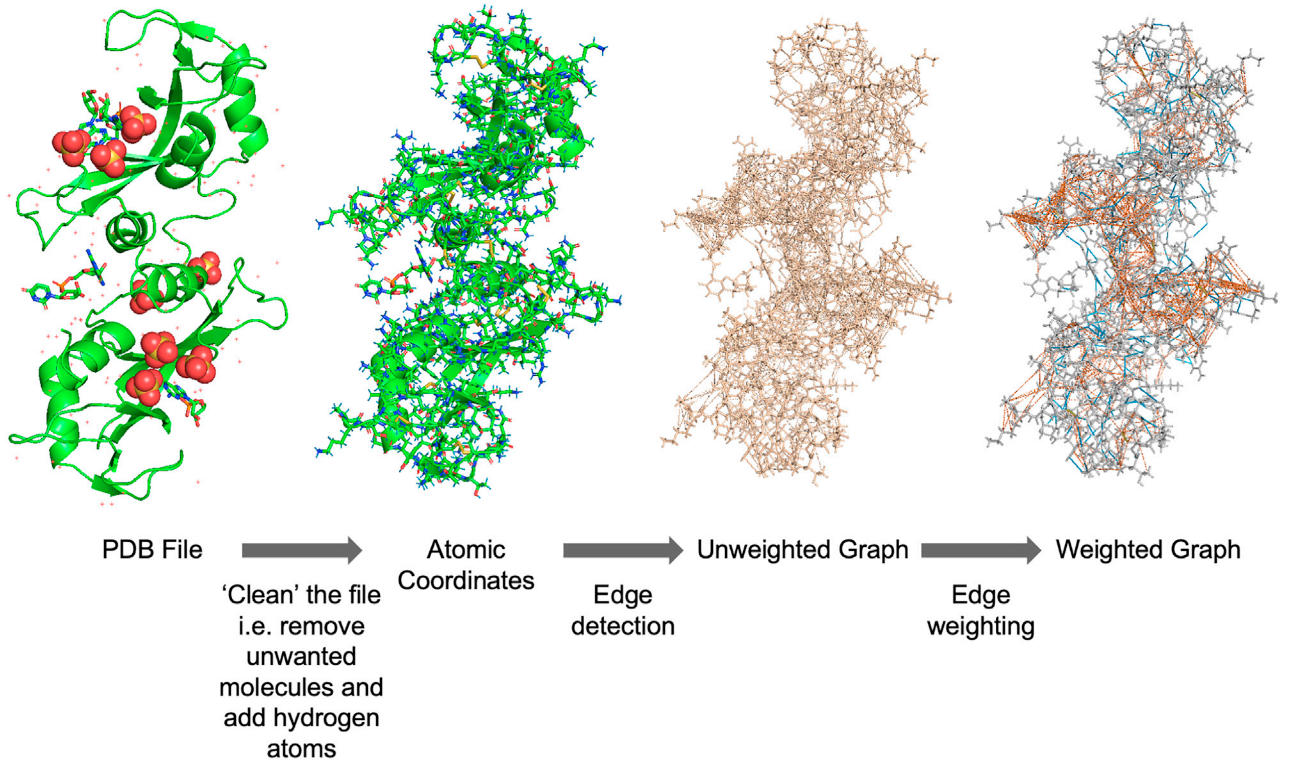

Figure 1. Atomistic graph construction
Main steps of the atomistic protein graph construction package, BagPype, using the structure of bovine seminal ribonuclease (PDB: 11BG)[66] as an example.

remain computationally efficient. It has been shown capable of predicting allosteric sites requiring only knowledge of orthosteric sites and ligands.[57] The method builds on the construction of an atomistic graph from a biomolecular structure with atoms described as nodes and bonds, whether covalent or noncovalent, as weighted edges (Figure 1). The resulting protein graph is analyzed with an edge-to-edge transfer matrix *M* (Method details), and the effect of fluctuations of an edge on any other edge is calculated and represented by a propensity score. Therefore, this approach enables the measurement of long-range coupling between bonds, which is crucial for allosteric signaling. This graph-theoretical model differs from all of the computational methods discussed above, except MD simulations, as it uses a fully atomistic representation of a protein that retains the physico-chemical details of a protein.[58,59] Despite keeping the atomistic details of the protein structure, the method is computationally efficient: by employing advances in algorithmic matrix theory,[60,61] the computation time scales approximately linearly with respect to the number of edges, which makes the method applicable to large and multimeric proteins[62,63] and high-throughput analysis in general. Furthermore, since there is no cutoff distance for interactions, both weak and long-range interactions within a protein can be captured by this model. Therefore, bond-to-bond propensity analysis presents a more cost-effective computational method to analyze proteins at the atomistic level and predict potential allosteric sites.

Bond-to-bond propensity analysis has successfully predicted 19 out 20 allosteric sites for a test set of 20 proteins[57] and showcased the allostery in aspartate carbamoyltransferase (ATCase) and the main protease of the severe acute respiratory syndrome coronavirus 2.[62,64] It has also been built into an efficient web application, ProteinLens, for the study of allostery.[65] To further benchmark this methodology and provide comparable insights into its performance across as diverse proteins as possible, we apply it here to two recently developed large, encompassing datasets, ASBench and CASBench. ASBench contains 235 allosteric sites,[34] and computational methods such as AlloPred,[50] AllositePro,[51] and SBSMMA[53] have made use of this dataset for method validation. However, it is important to note that some of these methods use only the chain of the protein that contains orthosteric and allosteric sites. This means they may potentially miss communication between the sites if the pathway involves multiple chains or the entire protein structure, as seen in multimeric proteins. We show in this work that bond-to-bond propensity analysis achieves overall higher accuracy in the ASBench dataset compared to the other methods using the same benchmarking dataset (see Table S1). We further tested bond-to-bond propensities with 314 structures of 33 proteins from a more recent dataset, CASBench, which contains proteins with multiple crystal structures.[35] We evaluated the allosteric site prediction performance of our method in these datasets based on the four statistical measures used in Amor et al.[57] and two new measures introduced in this work. Quantitative analysis of a given site with these measures provides mechanistic insights into the allosteric effects. The different scores can be exploited by data scientists, for example, working in digital chemistry to

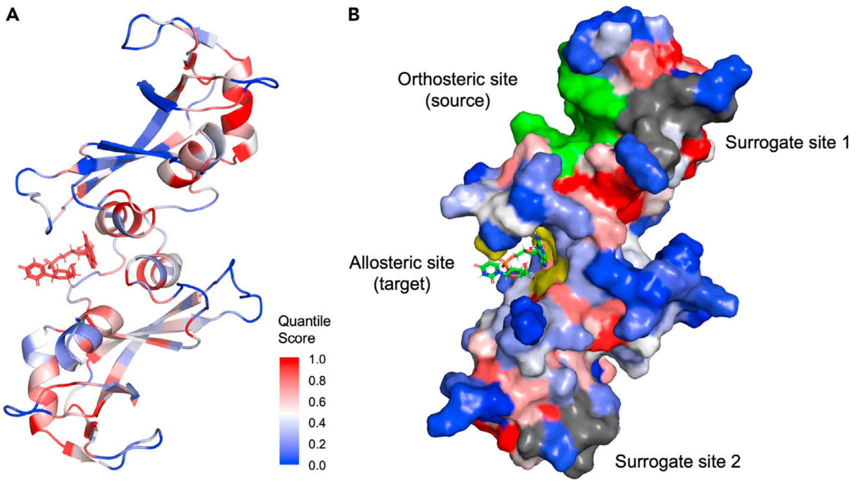

Figure 2. Bond-to-bond propensity analysis on the atomistic graph of bovine seminal ribonuclease (PDB: 11BG) where the orthosteric residues (green) are used as the perturbation source

(A) All residues are colored by residue QS (see legend) obtained from bond-to-bond propensity analysis.

(B) Surface representation of the protein structure colored by QS. Relevant sites are highlighted and labeled accordingly.

guide molecular design and synthesis to target specific sites on proteins for drug discovery through supervised learning and automation.

RESULTS

Bond-to-bond propensity analysis on the ASBench database

Proteins with annotated orthosteric residues, allosteric residues, and ligands were collected from the ASBench and ASD databases, as described in method details, which resulted in 118 structures of 113 distinct allosteric proteins. Bond-to-bond propensity analysis utilizes the orthosteric ligand as the perturbation source to mimic the ligand-binding event[57] and to identify regions on the protein that are functionally coupled to the orthosteric site. However, as orthosteric ligands are not available in structures from the ASBench database, the orthosteric site residues were selected as the perturbation source instead. For each protein, quantile scores (QSs), both intrinsic $(p_{b,\text{ allosteric site}}, p_{R,\text{ allosteric site}})$ and absolute $(p_b^{\text{ref}}, p_R^{\text{ref}})$, of all its bonds and residues can be calculated for the site(s) of interest. To assess the performance of the method and the significance of these calculated QSs, the allosteric site residues were used as the site(s) of interest and evaluated with six statistical measures (see method details).

We here exemplify the method on bovine seminal ribonuclease (PDB: 11BG),[66] where we used the orthosteric site residues (chain A: Asp14, Asn24, Asn27, Leu28, Asn94, and Cys95; chain B: Cys32 and Arg33) as the perturbation source. Figure 2 shows the propensity QS results mapped onto the protein structure, where blue (0) indicates a low and red (1) a high connectivity to the orthosteric site. The values obtained from the statistical measures for the allosteric residues (allosteric ligand excluded if present) are summarized in Table 1.

Based on the criteria described, the experimentally identified allosteric site can be detected with all six statistical measures. This process was conducted for all 118 proteins obtained from ASBench under two conditions: with and without the allosteric ligand in the structure. The results are shown in Figure 3.

In the presence of the allosteric ligand, the allosteric site is detected for 106 of 118 structures, according to at least one statistical measure, and for 81 of 118 structures, according to at least three statistical measures. When the allosteric ligand is removed from the protein structure and the same analysis is applied, the allosteric site is detected for 99 of 118 structures, according to at least one statistical measure, and for 69 of 118 structures, according to at least three statistical measures. The slight decrease in success rate is probably owing to the non-existence of interactions of the allosteric ligand with the allosteric site residues. Since these allosteric ligands are effective allosteric modulators of the corresponding protein, the binding of the allosteric ligand would strengthen the functional coupling of the allosteric site to the orthosteric site, which can be highlighted by the method. The average residue QS of the allosteric site for 109 of 118 structures decreased when the allosteric ligand was not present, and the QSs for the other nine structures only increased by less than 0.01, suggesting the same conclusion. Despite a lower success rate without the allosteric ligand, allosteric sites of 84% of the structures can be identified with only the knowledge of orthosteric site residues.

Prediction accuracy of bond-to-bond propensity analysis on the ASBench database

We focus here on the 12 structures with allosteric ligands where the allosteric site could not be detected by any of the measures. From those 12, the orthosteric residues of three structures (PDB: 1UXV, 2VD3, and 3QH0) reported in the ASD database are incorrect (they do not form a binding site), and those of one further structure (PDB: 2ATS) do not match with the data in ASBench. From the remaining eight, six structures (PDB: 1M8P, 3D2P, 3DC2, 3HQP, 3R1R, and 4HYW) obtained from the ASBench are only one part of a large and complex multimeric protein, where the effect of cooperativity might play a crucial role. Therefore, without the complete protein structure, the allosteric signaling cannot be detected. For example, it has been demonstrated with ATCase, a large dodecameric protein with six orthosteric sites, that only when at least three orthosteric sites are involved is allosteric behavior detected.[62] Since only one orthosteric site is reported in ASBench for these structures, this could explain the failure of identification of allosteric sites in these proteins when using only one orthosteric site as the perturbation source. From the remaining two structures, the G336V mutant of *Escherichia coli*, phosphoglycerate dehydrogenase

CellPress
OPEN ACCESS

Table 1. Results of bond-to-bond propensity analysis with six statistical measures for bovine seminal ribonuclease (PDB: 11BG)

Statistical measures	Results	Allosteric site detection
$\overline{p_{b,\ \text{allosteric site}}}$ [95% CI]	0.529 (> 0.495) [0.478, 0.495]	Success
$\overline{p_{R,\ \text{allosteric site}}}$ [95% CI]	0.665 (> 0.528) [0.522, 0.528]	Success
$P(p_{b,\ \text{allosteric site}} > 0.95)$	0.081 (> 0.05)	Success
$P(p_{R,\ \text{allosteric site}} > 0.95)$	0.125 (> 0.05)	Success
$\overline{p^{\text{ref}}_{b,\ \text{allosteric site}}}$	0.508 (> 0.5)	Success
$\overline{p^{\text{ref}}_{R,\ \text{allosteric site}}}$	0.780 (> 0.5)	Success

Note that for $\overline{p_{b,\ \text{allosteric site}}}$ and $\overline{p_{R,\ \text{allosteric site}}}$, the results need to be greater than the upper bound of the corresponding 95% confidence interval (95% CI), for $P(p_{b,\ \text{allosteric site}} > 0.95)$ and $P(p_{R,\ \text{allosteric site}} > 0.95)$, the results need to be greater than the expectation value of 0.05, and for $\overline{p^{\text{ref}}_{b,\ \text{allosteric site}}}$ and $\overline{p^{\text{ref}}_{R,\ \text{allosteric site}}}$, the results need to be greater than the expectation value of 0.5.

(PDB: 2PA3), displays a different allosteric mechanism, the flip flop mechanism,[67] which involves large-scale mechanical changes. Lastly, the human muscle glycogen phosphorylase (PDB: 1Z8D) contains two allosteric sites,[68] with only allosteric site 1 being detected, highlighted in red in Figure 4. This is due to the other site (highlighted in blue) being in close proximity to the orthosteric site, where the inhibition is achieved by blocking the entry channel to the orthosteric site.[69] Moreover, direct interactions, instead of functional coupling, occur if sites are close to the orthosteric sites, which is out of the scope of bond-to-bond propensity analysis, developed for allosteric, rather than direct, signaling detection.

Upon removing the allosteric ligands, allosteric sites of seven more structures could not be identified. For the structure of UDP-glucose dehydrogenase (PDB: 3PJG), ASBench has incorrect orthosteric residues reported (not forming a binding pocket), so a wrong perturbation source was used. Hemoglobin (PDB: 1B86) is a well-known protein with cooperativity underpinning its activity[70] that contains four orthosteric sites. As only one orthosteric site is reported in ASBench, the coupling of the allosteric site to this one site could not be detected as it might not be strong enough. Two structures (PDB: 3C1N and 3H6O) are large and complex multimeric proteins where again cooperativity would affect the results. The orthosteric sites and allosteric sites of the other three structures (PDB: 2W4I, 3MWB, and 4B1F), similar to those of 1Z8D above, are in close proximity. The allosteric effect is not mediated by functional coupling and is thus not revealed by propensity analysis.

It is worth noting that the allosteric sites are generally large in size based on the definition provided in the ASBench database (residues within 4 Å from the allosteric ligand). In the previous bovine seminal ribonuclease (PDB: 11BG) example, the allosteric site contains eight residues, but only four residues form direct interactions with the allosteric ligand. Therefore, these four residues are responsible for allosteric signaling as the direct interactions connecting the ligand and the protein are essentially where the perturbation starts (see Table 2).

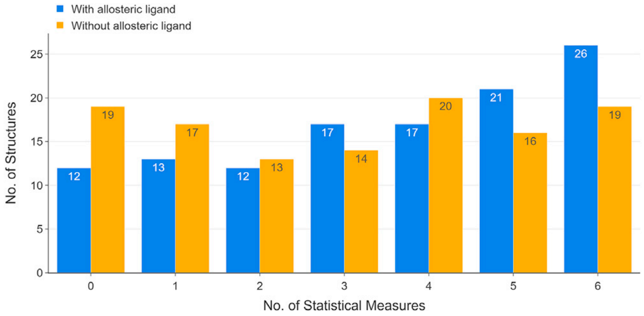

Figure 3. Summary of allosteric site detection results for 118 structures in the ASBench database

Propensity analysis was conducted for all 118 structures under two conditions: (1) with allosteric ligand in the protein structure (blue) and (2) without allosteric ligand in the protein structure (orange). The *x*-axis represents the number of statistical measures that successfully identify the allosteric site. Each bar indicates the number of protein structures of which the allosteric sites can be detected by a certain number of statistical measures shown on the *x*-axis. Take the last two bars as an example: the allosteric site(s) can be detected using all six statistical measures for 26 proteins structures with the presence of allosteric ligand (blue bar). When the allosteric ligand is removed from the structures, allosteric site(s) of 19 structures can be identified with all six measures (orange bar). Detailed data can be found in Tables S3 and S4.

$\overline{p_{b,\ \text{allosteric site}}}$ does not change, whereas $\overline{p_{R,\ \text{allosteric site}}}$ decreases slightly when only four allosteric residues were scored. However, the drop in mean QS and the 95% confidence interval calculated from the 1,000 surrogate sites indicates that the allosteric site becomes more significant compared with other similar sites on the protein. The increase of values for the other four measures complements this argument. Therefore, defining the allosteric site with the four interacting residues leads to better detection of the allosteric site, and one needs to take note that actual results may be buried by the definition of a large allosteric site. Hence, it is important to characterize the allosteric site and include relevant residues properly, which presents an ongoing problem.[71]

Similarly, not all residues in the orthosteric site defined in the database interact with the orthosteric ligand or support its binding. Due to the absence of orthosteric ligands in the structures from the ASBench database, comparisons between using the orthosteric site residues and the orthosteric ligand as perturbation source cannot be achieved.

Bond-to-bond propensity analysis on the CASBench database

314 structures of 33 allosteric proteins with orthosteric ligands and description of orthosteric and allosteric residues were collected from the CASBench database. As seen in the ASBench data analysis above, the presence of the allosteric ligand strengthens the coupling to the orthosteric site and makes the result biased toward successful detection of the allosteric site. Hence, the allosteric ligand (if present in the structure) is removed when carrying out bond-to-bond propensity analysis for the CASBench database.

Bond-to-bond propensity analysis was conducted for these 314 structures using the orthosteric ligand or orthosteric site residues (with orthosteric ligand removed) as the perturbation

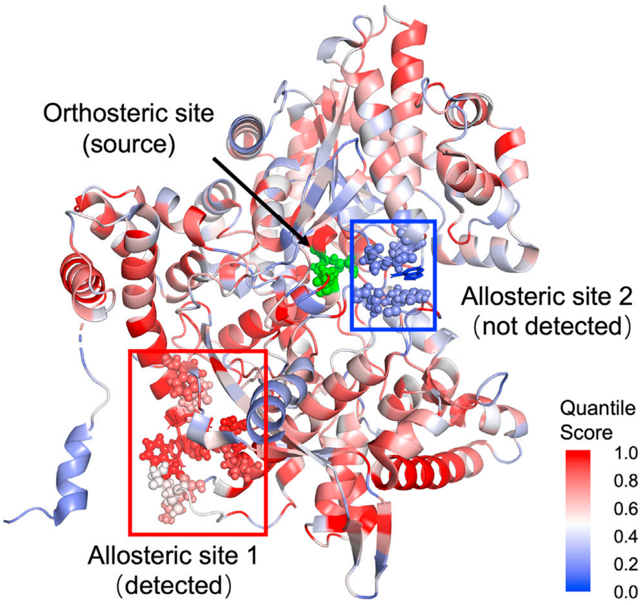

Figure 4. Structure of human muscle glycogen phosphorylase (PDB: 1Z8D)
The orthosteric (green) and two allosteric (circled in blue and red) site residues are highlighted as spheres.

Table 2. Results of bond-to-bond propensity analysis with six statistical measures for bovine seminal ribonuclease (PDB: 11BG)

Statistical measures	Results (8 allosteric residues)	Results (4 allosteric residues)
$\overline{p_{b,\text{ allosteric site}}}$ [95% CI]	0.529 (> 0.495) [0.478, 0.495]	0.529 (> 0.495) [0.475, 0.495]
$\overline{p_{R,\text{ allosteric site}}}$ [95% CI]	0.665 (> 0.528) [0.522, 0.528]	0.659 (> 0.501) [0.494, 0.501]
$P(p_{b,\text{ allosteric site}} > 0.95)$	0.081 (> 0.05)	0.106 (> 0.05)
$P(p_{R,\text{ allosteric site}} > 0.95)$	0.125 (> 0.05)	0.25 (> 0.05)
$\overline{p^{\text{ref}}_{b,\text{ allosteric site}}}$	0.508 (> 0.5)	0.510 (> 0.5)
$\overline{p^{\text{ref}}_{R,\text{ allosteric site}}}$	0.780 (> 0.5)	0.808 (> 0.5)

Note that for $\overline{p_{b,\text{ allosteric site}}}$ and $\overline{p_{R,\text{ allosteric site}}}$, the results need to be greater than the upper bound of the corresponding 95% confidence interval (95% CI), for $P(p_{b,\text{ allosteric site}} > 0.95)$ and $P(p_{R,\text{ allosteric site}} > 0.95)$, the results need to be greater than the expectation value of 0.05, and for $\overline{p^{\text{ref}}_{b,\text{ allosteric site}}}$ and $\overline{p^{\text{ref}}_{R,\text{ allosteric site}}}$, the results need to be greater than the expectation value of 0.5.

source in two separate runs. When multiple orthosteric ligands or sites are present, all of them were used as the perturbation source. Moreover, when there are multiple allosteric sites in the protein structure, each of them is investigated separately with the six statistical measures, and the average value for each of the measures is used to decide whether the allosteric sites can be detected for the protein. Table 3 summarizes the results for yeast chorismate mutase (PDB: 3CSM),[72] an example of a system with two allosteric sites.

It is observed in some cases that some of the allosteric sites of the protein can be detected by a particular measure, whereas the other sites cannot be detected ($P(p_{b,\text{ allosteric site}} > 0.95)$ in this case). Therefore, the criteria used here are stringent and would be effective and meaningful in assessing the performance of bond-to-bond propensity analysis; the performance summary is shown in Figure 5.

When the orthosteric ligand is selected as the perturbation source, the allosteric site is detected for 308 of 314 structures (32 of 33 proteins), according to at least one statistical measure. When using the orthosteric site residues as the perturbation source, the allosteric site is detected for 304 of 314 structures (32 of 33 proteins), according to at least one statistical measure. It is observed that, in general, the allosteric site of a protein structure can be identified with more statistical measures when the orthosteric ligand is set as the perturbation source.

If the orthosteric ligand is selected as the perturbation source, the source bonds include the weak bonds formed by the ligand and the surrounding residues. The orthosteric site includes all residues within 5 Å of the orthosteric ligand.[35] Therefore, the number of source bonds is much lower compared with when using the entire orthosteric site residues as the perturbation source. The different and better results obtained by using the ligand as the perturbation source suggest that the allosteric site is closely coupled to the ligand-binding event at the orthosteric site. Although successful allosteric site detection is achieved by fewer statistical measures using the whole orthosteric site as the perturbation source, the method still succeeds in identifying allosteric sites for more than 96% of the 314 structures. Combined with the results from analyzing the ASBench database, for which orthosteric residues are used as the perturbation source, the results indicate that propensity analysis reveals the intrinsic coupling of the allosteric site to the region where the orthosteric binding occurs. Using the orthosteric ligand as the perturbation source allows a more accurate detection of allosteric sites. However, if there is no structure containing the orthosteric ligand, the approximate site containing orthosteric residues would still be a good choice to uncover distant sites coupled to the region and provide guidance on allosteric site detection.

Prediction accuracy of bond-to-bond propensity analysis on the CASBench database

We focus here on the six structures for which the allosteric site cannot be detected by any of the measures when using orthosteric ligands as the perturbation source. One of them (PDB: 4R1R) is ribonucleotide reductase protein R1 (CAS0047). It is a large and complex multimeric protein, and only one orthosteric site is reported in the CASBench database. Hence, the effect of cooperativity could affect the performance of propensity analysis as previously discussed. Another two structures (PDB: 1FUQ and 1KQ7) are two out of the four structures of fumarase (CAS0085). This is also a complex multimeric protein where bond-to-bond propensity analysis may not perform well if not all orthosteric ligands are present. The remaining three structures are epoxide hydrolase (CAS0002) (PDB: 5AIA, 5ALN, and 5ALT). We analyzed 28 structures of epoxide hydrolase in total, each with a different orthosteric ligand. Hence, different ligands, even when binding at the same orthosteric site, exert different perturbation effects on the protein.

Table 3. Results of bond-to-bond propensity analysis with six statistical measures and averaging for yeast chorismate mutase (PDB: 3CSM)

Statistical measures	Results	Average	Allosteric site detection
$\overline{p_{b,\text{ allosteric site}}}$ [95% CI]	Site 1: 0.518 (> 0.505) [0.499, 0.505]; Site 2: 0.527 (> 0.505) [0.498, 0.503]	0.522 (> 0.505) [0.499, 0.505]	Success
$\overline{p_{R,\text{ allosteric site}}}$ [95% CI]	Site 1: 0.560 (> 0.531) [0.529, 0.531]; Site 2: 0.598 (> 0.530) [0.527, 0.530]	0.579 (> 0.531) [0.529, 0.531]	Success
$P(p_{b,\text{ allosteric site}} > 0.95)$	Site 1: 0.048 (< 0.05); Site 2: 0.060 (> 0.05)	0.054 (> 0.05)	Success
$P(p_{R,\text{ allosteric site}} > 0.95)$	Site 1: 0.056 (> 0.05); Site 2: 0.056 (> 0.05)	0.056 (> 0.05)	Success
$\overline{p^{\text{ref}}_{b,\text{ allosteric site}}}$	Site 1: 0.491 (< 0.5); Site 2: 0.495 (< 0.5)	0.493 (< 0.5)	Failure
$\overline{p^{\text{ref}}_{R,\text{ allosteric site}}}$	Site 1: 0.586 (> 0.5); Site 2: 0.607 (> 0.5)	0.596 (> 0.5)	Success

The two allosteric sites were scored separately based on the six metrics separately, and the average score was used to assess whether the allosteric sites of yeast chorismate mutase can be detected by each measure. Note that for $\overline{p_{b,\text{ allosteric site}}}$ and $\overline{p_{R,\text{ allosteric site}}}$, the results need to be greater than the upper bound of the corresponding 95% confidence interval (95% CI), for $P(p_{b,\text{ allosteric site}} > 0.95)$ and $P(p_{R,\text{ allosteric site}} > 0.95)$, the results need to be greater than the expectation value of 0.05, and for $\overline{p^{\text{ref}}_{b,\text{ allosteric site}}}$ and $\overline{p^{\text{ref}}_{R,\text{ allosteric site}}}$, the results need to be greater than the expectation value of 0.5.

When orthosteric residues were used as the perturbation source, the allosteric sites of two structures (PDB: 1LLD and 1LTH) of L-lactate dehydrogenase (CAS0028) were not identified. This can be partly explained by the changed perturbation effects as the allosteric sites were identified when sourcing from the orthosteric ligands. In CASBench, the orthosteric sites include residues within 5 Å from the orthosteric ligands, which leads to a large region as the perturbation source. This shows that the specific ligand-site interactions are crucial for accurate allosteric site detection. This is consistent with the overall trend since it has been shown above that successful allosteric site detection is achieved by more statistical measures using the orthosteric ligand as the perturbation source. Moreover, allosteric sites of another eight structures were not detected when only using the orthosteric site residues as the perturbation source. This further strengthens the idea that the method is sensitive to specific interactions between the ligand and the protein and holds the potential to evaluate the performance of different ligands in the orthosteric site.

DISCUSSION

Allosteric sites are of great interest in understanding biological function as well as in drug targeting, but they are difficult to predict and, in general, poorly understood. They are usually discovered serendipitously and require experimental verification. Two recently introduced allosteric protein databases, ASBench[34] and CASBench,[35] aim to collect available information on known allosteric sites and are hence excellent benchmarking tools for promising computational approaches. To test the capability of bond-to-bond propensity analysis, a recently developed method that was shown to be able to predict allosteric sites, we deployed the method to both databases, which, after cleaning, provided 432 protein structures for analysis.

An important part of this process is the scoring of the target sites. In addition to previously used scoring measures, we introduced two additional statistical measures, namely the average reference residue QS of the allosteric residues, $\overline{p^{\text{ref}}_{R,\text{ allosteric site}}}$, and the proportion of allosteric residues with a QS greater than 0.95, $P(p_{R,\text{ allosteric site}} > 0.95)$. The first measures the absolute propensities of residues in the allosteric site compared with the Structural Classification of Proteins (SCOP) reference set, and the second counts the number of high scoring residues in the allosteric site. These two measures complement the existing four metrics and enable thorough analysis of the significance of the QS computed from bond-to-bond propensity analysis. In the previous benchmarking against ASBench, we only applied the four measures from Amor et al. and identified allosteric sites for 102 out of 118 structures.[65] The additional measures enable us to identify allosteric sites of four more protein structures. The reason we have six different measures roots from the unclear definition of allosteric mechanisms. Some may argue that the ligand-binding event at the allosteric site causes a global conformational change of the protein that leads to the allosteric effect, but some attribute the effect to signaling between the orthosteric and the allosteric sites. $\overline{p_{b,\text{ allosteric site}}}$ and $\overline{p_{R,\text{ allosteric site}}}$ evaluate the intrinsic coupling strength of the entire allosteric site to the orthosteric site. $P(p_{b,\text{ allosteric site}} > 0.95)$ and $P(p_{R,\text{ allosteric site}} > 0.95)$ focus more on the critical bonds and residues responsible for allosteric signaling. Lastly, $\overline{p^{\text{ref}}_{b,\text{ allosteric site}}}$ and $\overline{p^{\text{ref}}_{R,\text{ allosteric site}}}$ further confirm the coupling between the allosteric and orthosteric sites. Unlike most of the present computational methods that use one score to rank cryptic sites and predict the allosteric site, using multiple scores in the prediction of allosteric sites by considering different aspects of allosteric effects is practical. This can also be adopted by researchers in this field to further benchmark and improve on the quantitative analysis of a target site of a protein. This expands the scope of future work on how to best use the different scores in predicting allosteric sites as well as sheds light on potential allosteric mechanisms involved within the protein through carefully examining the proteins tested in this work and the corresponding scores from the six measures.

Benchmarking datasets of allosteric proteins, namely the ASBench and the CASBench databases, were used for analysis. For structures in ASBench, the orthosteric residues were used as the perturbation source. With the presence of the allosteric ligand, the allosteric site is identified for 106 of 118 (89.8%) structures and the allosteric site is detected for 99 of 118 (83.9%)

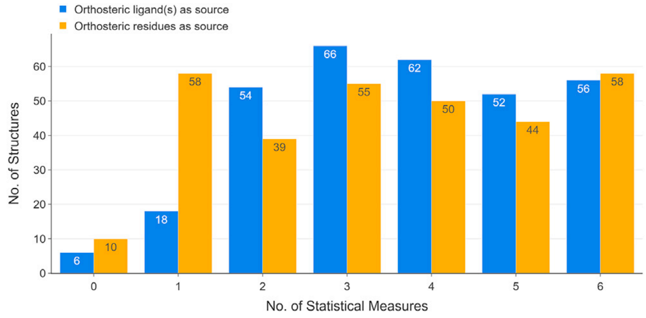

Figure 5. Summary of allosteric site detection results for 314 structures in the CASBench database

Propensity analysis was conducted for all 314 structures under two conditions: (1) using orthosteric ligand(s) as the perturbation source (blue) and (2) using orthosteric residues (removed orthosteric ligands) as the perturbation source (orange). The *x*-axis represents the number of statistical measures that successfully identify the allosteric site. Each bar indicates the number of protein structures of which the allosteric sites can be detected by a certain number of statistical measures shown on the *x*-axis. Take the last two bars as an example: the allosteric site(s) can be detected using all six statistical measures for 56 proteins structures when using the orthosteric ligand(s) as the perturbation source. When using the orthosteric site residues as the perturbation source, allosteric site(s) of 58 structures can be identified with all six measures. Detailed data can be found in Tables S6 and S7.

structures when the allosteric ligand is removed, according to at least one statistical measure. Despite the strengthening of functional coupling of the allosteric site to the orthosteric site by the allosteric ligand, propensity analysis is still able to reveal the intrinsic connectivity between the two sites. For the CASBench database, we conducted our analysis sourced from the orthosteric ligands or the orthosteric residues and managed to detect the allosteric sites according to at least one statistical measure for 308 of 314 (98.1%) structures (32 of 33 proteins) and for 304 of 314 (96.8%) structures (32 of 33 proteins), respectively. The allosteric site of a protein structure can be identified with more statistical measures when choosing the orthosteric ligand as the perturbation source. This observation suggests that using the ligand as the perturbation source confers the perturbation effect of the binding event more accurately. However, if the information on the orthosteric substrate is not available, it is viable to select the orthosteric residues as the perturbation source.

Four existing computational methods have been benchmarked using the allosteric protein data from ASBench and ASD. Since the protocols for protein structure selection and collection of relevant site information are different for each method, a direct comparison cannot be fully achieved. The prediction accuracies of AllositePro,[51] AlloPred,[50] and PARS[48] are 51.7%, 59%, and 65%, respectively, while Tee et al.[53] did not report the prediction accuracy of SBSMMA. Bond-to-bond propensity analysis outperforms these methods with a prediction accuracy of 84% when benchmarked against 118 structures from the ASBench dataset.

The results presented here strengthen confidence in allosteric site identification predicted by bond-to-bond propensity, which coupled with the efficiency of the method make it an attractive approach. Generally, the definition of orthosteric and allosteric residues, which would significantly affect the size and residues involved, plays an essential part when evaluating allosteric site prediction methods and was also highlighted for bond-to-bond propensity analysis. Finally, more detailed analysis would be usually required in cases where the allosteric site and the orthosteric site are in very close proximity, to elucidate the effect of cooperativity in large and complex multimeric proteins or the role of structural water molecules, which could still be possible given the computational efficiency of the approach. The introduction of the statistical measures coupled with the availability of large datasets and the efficiency of computing bond-to-bond propensity taken together strengthens our understanding of allostery and builds the groundwork to a more targeted and data-driven allosteric drug design.

EXPERIMENTAL PROCEDURES

Resource availability

Lead contact

Further information and requests for resources should be directed to and will be fulfilled by the lead contact, Sophia N. Yaliraki (s.yaliraki@imperial.ac.uk).

Data and code availability

All protein structures used in this project and results obtained using bond-to-bond propensity are deposited at figshare with https://doi.org/10.6084/m9.figshare.16940317.v1. The method can be accessed via the ProteinLens webserver.[65]

Materials availability

The authors declare that no materials were generated or used during this study.

Allosteric protein datasets

The ASBench database

235 X-ray crystal structures of allosteric proteins were downloaded from the ASBench database. Experimentally determined orthosteric and allosteric site residues for these proteins were attained from ASD Release 4.10.[73] The data was further processed to exclude entries without orthosteric site information or incomplete structures. The resulting 118 structures were all analyzed by bond-to-bond propensity. Details can be found in Table S2. Note that results on the first four of the six scoring measures were first reported in the supplementary information of Mersmann et al.[65] without any analysis.

The CASBench database

X-ray crystal structures containing various orthosteric and allosteric ligands of 91 allosteric proteins in PDB format were downloaded from the CASBench website together with the corresponding experimentally determined orthosteric and allosteric site residues. This data was further processed to exclude incomplete structures, and the resulting 314 structures of 33 distinct proteins were used for bond-to-bond propensity analysis. The proteins in CASBench are labeled with CAS ID, and the list of proteins with corresponding CAS ID used in this work can be found in Table S5.

Method details

Construction of the atomistic protein graph

Bond-to-bond propensity analysis starts by constructing a weighted atomistic graph using the three-dimensional coordinates of the atoms of the protein in the PDB files. Atoms are represented by nodes, and interactions (covalent and non-covalent) between the atoms are represented by edges. The weights of edges correspond to the interaction energies between the atoms with weights derived from relevant interatomic potentials. An in-depth procedure for the atomistic protein graph construction has been described in Delmotte et al. and Amor et al.[58,59] In this work, Biochemical, atomistic graph construction software in Python for proteins (BagPype)[65,74] was used to construct the atomistic protein graph, and Figure 1 illustrates the main features of this process using bovine seminal ribonuclease (PDB: 11BG)[66] as an example. The crystal structures in the PDB files are cleaned by removing water molecules and unwanted ligands followed by adding hydrogen atoms using Reduce (v.3.23),[75] which is incorporated in BagPype. Covalent bonds are weighted using standard bond energies.[76] The weighting of π-π stacking, hydrophobic interaction, hydrogen bonding,

and electrostatic interactions is done based on potentials in Hunter and Sanders,[77] Lin et al.,[78] Mayo et al.,[79] and Jorgensen and Tirado-Tives,[80] respectively. The weighted graph is then converted to an $N \times N$ adjacency matrix, where N is the number of nodes (atoms).

Bond-to-bond propensities

Bond-to-bond propensity was first introduced in Amor et al.[57] and further discussed in Hodges et al.,[62] so it is only briefly summarized here. The edge-to-edge transfer matrix M was introduced to study non-local edge-coupling in graphs,[81] and an alternative interpretation of M is employed to analyze the atomistic protein graph. The element M_{ij} describes the effect that a perturbation at edge i has on edge j. M is given by

$$M = \frac{1}{2} W B^T L^{\dagger} B \quad \text{(Equation 1)}$$

where B is the $n \times m$ incidence matrix for the atomistic protein graph with n nodes and m edges; $W = \mathrm{diag}(w_{ij})$ is an $m \times m$ diagonal matrix that possesses all edge interaction energies with w_{ij} as the weight of the edge connecting nodes i and j, i.e., the bond energy between the atoms. $L^{\dagger}$ is the pseudo-inverse of the weighted graph Laplacian matrix L.[82] L, which defines the diffusion dynamics on the energy-weighted graph,[83] is defined as follows:

$$L_{ij} = \begin{cases} -w_{ij}, & i \neq j \\ \sum_j w_{ij}, & i = j \end{cases} \quad \text{(Equation 2)}$$

To evaluate the effect of perturbations from a group of bonds b', which belong to the orthosteric ligand or the orthosteric site residues (i.e., the perturbation source), on a bond b anywhere else in the protein, we calculate the following:

$$\prod_b^{raw} = \sum_{b' \in source} |M_{bb'}| \quad \text{(Equation 3)}$$

This is the raw propensity of an individual bond that reflects how strongly the bond is coupled to the perturbation source. As different proteins contain different numbers of bonds, the raw propensity is normalized and the bond propensity is defined as follows:

$$\prod_b = \frac{\prod_b^{raw}}{\sum_b \prod_b^{raw}} \quad \text{(Equation 4)}$$

The residue propensity is then defined as the sum of normalized bond propensities of all the bonds of a residue, R:

$$\prod_R = \sum_{b \in R} \prod_b \quad \text{(Equation 5)}$$

Quantile regression

Bond and residue propensities naturally decrease as the distance of the bond or residue from the perturbation source increases. To determine the bonds and residues that are significant, bond and residue propensities at a similar distance from the perturbation source are compared using conditional quantile regression (QR).[84] The distance of a bond b from the perturbation source is defined as the minimum distance, d_b, between b and any bond of the perturbation source:

$$d_b = \min_{b' \in source} |\mathbf{x}_b - \mathbf{x}_{b'}|, \quad \text{(Equation 6)}$$

where the vector $\mathbf{x}_b$ contains the cartesian coordinates of the midpoint of bond b. As propensity $\prod_b$ decays exponentially with distance d, a linear model for the logarithm of the propensities is adopted to solve the QR minimization problem:

$$\hat{\beta}_b^{protein}(p) = \underset{(\beta_{b,0},\beta_{b,1})}{\mathrm{argmin}} \sum_b^{protein} \rho_p \left(log\left(\prod_b\right) - (\beta_{b,0} + \beta_{b,1} d_b) \right), \quad \text{(Equation 7)}$$

where $\rho_p(\cdot)$ is the tilted absolute value function,

$$\rho_p(y) = |y(p - 1(y < 0))|, \quad \text{(Equation 8)}$$

p is the quantile, and $1(\cdot)$ is the indicator function. The optimized model $\hat{\beta}^{protein} = (\hat{\beta}_{b,0}^{protein}(p), \hat{\beta}_{b,1}^{protein}(p))$ describes the sum of the quantiles of the propensities for all bonds in the protein. The bond quantile score of bond b with propensity $\prod_b$ at distance d_b from the perturbation source can be calculate by finding the quantile p_b such that

$$p_b = \underset{p \in [0,1]}{\mathrm{argmin}} \left| log\left(\prod_b\right) - \left(\hat{\beta}_{b,0}^{protein}(p) + \hat{\beta}_{b,1}^{protein}(p) d_b\right) \right| \quad \text{(Equation 9)}$$

The residue quantile score of residue R is defined similarly by using the residue propensity as shown in Equation 5 and the distance d_p, which is the minimum distance between the atoms of a residue and those of the perturbation source. Therefore,

$$\hat{\beta}_R^{protein}(p) = \underset{(\beta_{R,0},\beta_{R,1})}{\mathrm{argmin}} \sum_R^{protein} \rho_p \left(log\left(\prod_R\right) - (\beta_{R,0} + \beta_{R,1} d_R) \right), \quad \text{(Equation 10)}$$

and

$$p_R = \underset{p \in [0,1]}{\mathrm{argmin}} \left| log\left(\prod_R\right) - \left(\hat{\beta}_{R,0}^{protein}(R) + \hat{\beta}_{R,1}^{protein}(p) d_R\right) \right| \quad \text{(Equation 11)}$$

are used to calculate the residue quantile score.

Statistical evaluation of allosteric bond and residue quantile scores

Four statistical measures have been used to evaluate the significance of the QS by Amor et al.[57] and were employed in this project, as listed below:

1. The average bond QS of the allosteric site:

$$\overline{p_{b,\ \text{allosteric site}}} = \frac{\sum_{b\ \in\ \text{allosteric site}} p_b}{N_{b,\ \text{allosteric site}}} \quad \text{(Equation 12)}$$

where N_b, allosteric site is the number of bonds in the allosteric site.

2. The average residue QS of the allosteric site:

$$\overline{p_{R,\ \text{allosteric site}}} = \frac{\sum_{R\ \in\ \text{allosteric site}} p_R}{N_{R,\ \text{allosteric site}}} \quad \text{(Equation 13)}$$

where $N_{R,\ \text{allosteric site}}$ is the number of residues in the allosteric site.

3. The proportion of bonds in the allosteric site with bond QS greater than 0.95,

i.e., $P(p_{b,\ \text{allosteric site}} > 0.95)$.

4. The average reference bond QS of the allosteric site:

$$\overline{p^{ref}_{b,\ \text{allosteric site}}} = \frac{\sum^{ref}_{b\ \in\ \text{allosteric site}} p^{ref}_b}{N_{b,\ \text{allosteric site}}} \quad \text{(Equation 14)}$$

where $N_{b,\ \text{allosteric site}}$ is the number of bonds in the allosteric site.

For the purpose of complementing these previous measures and to investigate more aspects of allosteric site detection, two additional measures were introduced in this work:

5. The proportion of residues in the allosteric site with residue QS greater than 0.95,

i.e., $P(p_{R,\ \text{allosteric site}} > 0.95)$.

6. The average reference residue QS of the allosteric site:

$$\overline{p^{ref}_{R,\ \text{allosteric site}}} = \frac{\sum^{ref}_{R\ \in\ \text{allosteric site}} p^{ref}_R}{N_{R,\ \text{allosteric site}}} \quad \text{(Equation 15)}$$

where $N_{R,\ \text{allosteric site}}$ is the number of residues in the allosteric site.

If the functional coupling is the result of a cumulative effect of the whole allosteric site, an accurate measure of the allosteric propensity would be the average QS of all bonds or residues in the allosteric site. Hence, measures 1, 2, 4, and 6 would be able to uncover the cumulative effect at both the

bond and residue level for both the intrinsic propensities of the protein and the absolute propensities comparing with the SCOP reference set.[57] It is also possible that only a few bonds or residues with high QS in the allosteric site are responsible for the functional coupling to the orthosteric site, while other allosteric bonds or residues are associated with structural and energetics aspects of allosteric ligand binding. Measures 3 and 5 are able to detect the proportion of those high scoring bonds. This is because QS is uniformly distributed, and the bonds with QS greater than 0.95 belong to the top 5% of all the bonds in the protein.

To assess the significance of the average bond and residue QS $\overline{p_{b,\text{ allosteric site}}}$ and $\overline{p_{R,\text{ allosteric site}}}$, structural bootstrap is used to sample random surrogate sites from the same protein. These surrogate sites need to follow two structural rules: (1) the number of residues is equal to the number of residues in the allosteric site, and (2) the diameter (maximum distance between any two atoms in the site) is smaller than that of the allosteric site. For each protein, 1,000 surrogate sites are generated, and the average bond and residue QS $\langle\overline{p_{b,\text{ site}}}\rangle_{\text{surrogate sites}}$ and $\langle\overline{p_{R,\text{ site}}}\rangle_{\text{surrogate sites}}$ of these sites are calculated. The scores are compared with those of the allosteric sites $(\overline{p_{b,\text{ allosteric site}}}$ and $\overline{p_{R,\text{ allosteric site}}})$. A 95% confidence interval is obtained for each protein to assess the statistical significance by using bootstrap with 10,000 resamples with replacement.[85] Figure 2 illustrates the process using bovine seminal ribonuclease (PDB: 11BG)[66] as an example. If the average QS, whether bond or residue of the allosteric residues, is greater than the upper bound of the 95% confidence interval, the allosteric site is assumed to be detected according to the corresponding statistical measure. The proportion of both bonds and residues of the allosteric residues with a QS greater than 0.95 $(P(p_{b,\text{ allosteric site}} > 0.95)$ and $P(p_{R,\text{ allosteric site}} > 0.95))$ is then calculated. If the proportion exceeds the expected proportion of 0.05, the allosteric site is classified as identified. Lastly, the average reference bond and residue QS of the allosteric residues $(\overline{p^{\text{ref}}_{b,\text{ site}}}$ and $\overline{p_{R,\text{ site}}})$ are computed, and a value greater than 0.5 (the expected value) suggests that the allosteric site is uncovered.

SUPPLEMENTAL INFORMATION

Supplemental information can be found online at https://doi.org/10.1016/j.patter.2021.100408.

ACKNOWLEDGMENTS

We acknowledge helpful discussions with Florian Song, Ching Ching Lam, and Jerzy Pilipczuk. This work was funded by the President's PhD Scholarships, Imperial College London, to N.W.. L.S. acknowledges funding from a Wellcome Trust studentship [grant number 215360/Z/19/Z]. N.W. and S.N.Y. acknowledge funding from the EPSRC award EP/N014529/1 supporting the EPSRC Centre for Mathematics of Precision Healthcare.

AUTHOR CONTRIBUTIONS

N.W., L.S., and S.N.Y. conceived the study. N.W. performed the computations and created the figures, and all authors analyzed the data and wrote the manuscript.

DECLARATION OF INTERESTS

The authors declare no competing interests.

Received: August 18, 2021
Revised: September 6, 2021
Accepted: November 15, 2021
Published: December 9, 2021

REFERENCES

1. Casem, M.L. (2016). Chapter 3 - Proteins. Case Studies in Cell Biology (Academic Press), pp. 23–71. https://doi.org/10.1016/B978-0-12-801394-6.00003-8.
2. Gonzalez, M.W., and Kann, M.G. (2012). Chapter 4: protein interactions and disease. PLoS Comput. Biol. *8*, 1–11. https://doi.org/10.1371/journal.pcbi.1002819.
3. Santos, R., Ursu, O., Gaulton, A., Bento, A.P., Donadi, R.S., Bologa, C.G., Karlsson, A., Al-Lazikani, B., Hersey, A., Oprea, T.I., and Overington, J.P. (2017). A comprehensive map of molecular drug targets. Nat. Rev. Drug Discov. *16*, 19–34. https://doi.org/10.1038/nrd.2016.230.
4. Abdel-Magid, A.F. (2015). Allosteric modulators: an emerging concept in drug discovery. ACS Med. Chem. Lett. *6*, 104–107. https://doi.org/10.1021/ml5005365.
5. Grover, A.K. (2013). Use of allosteric targets in the discovery of safer drugs. Med. Principles Pract. *22*, 418–426. https://doi.org/10.1159/000350417.
6. Traxler, P., and Furet, P. (1999). Strategies toward the design of novel and selective protein tyrosine kinase inhibitors. Pharmacol. Ther. *82*, 195–206. https://doi.org/10.1016/S0163-7258(98)00044-8.
7. Munita, J.M., and Arias, C.A. (2016). Mechanisms of antibiotic resistance. Microbiol. Spectr. *4*, 0016–2015. https://doi.org/10.1128/microbiolspec.VMBF-0016-2015.
8. Li, W., Atkinson, G.C., Thakor, N.S., Allas, U., Lu, C.-c., Chan, K.-Y., Tenson, T., Schulten, K., Wilson, K.S., Hauryliuk, V., and Frank, J. (2013). Mechanism of tetracycline resistance by ribosomal protection protein Tet(O). Nat. Commun. *4*, 1477. https://doi.org/10.1038/ncomms2470.
9. Dönhöfer, A., Franckenberg, S., Wickles, S., Berninghausen, O., Beckmann, R., and Wilson, D.N. (2012). Structural basis for TetM-mediated tetracycline resistance. Proc. Natl. Acad. Sci. U S A *109*, 16900–16905. https://doi.org/10.1073/pnas.1208037109.
10. Hooper, D.C. (2002). Fluoroquinolone resistance among Gram-positive cocci. Lancet Infect. Dis. *2*, 530–538. https://doi.org/10.1016/S1473-3099(02)00369-9.
11. Leclercq, R. (2002). Mechanisms of resistance to macrolides and lincosamides: nature of the resistance elements and their clinical implications. Clin. Infect. Dis. *34*, 482–492. https://doi.org/10.1086/324626.
12. Hiramatsu, K., Ito, T., Tsubakishita, S., Sasaki, T., Takeuchi, F., Morimoto, Y., Katayama, Y., Matsuo, M., Kuwahara-Arai, K., Hishinuma, T., and Baba, T. (2013). Genomic basis for methicillin resistance in *Staphylococcus aureus*. Infect. Chemother. *45*, 117–136. https://doi.org/10.3947/ic.2013.45.2.117.
13. Wodak, S.J., Paci, E., Dokholyan, N.V., Berezovsky, I.N., Horovitz, A., Li, J., Hilser, V.J., Bahar, I., Karanicolas, J., Stock, G., et al. (2019). Allostery in its many disguises: from theory to applications. Structure *27*, 566–578. https://doi.org/10.1016/j.str.2019.01.003.
14. Peracchi, A., and Mozzarelli, A. (2011). Exploring and exploiting allostery: models, evolution, and drug targeting. Biochim. Biophys. Acta *1814*, 922–933. https://doi.org/10.1016/j.bbapap.2010.10.008.
15. Kenakin, T., and Miller, L.J. (2010). Seven transmembrane receptors as shapeshifting proteins: the impact of allosteric modulation and functional selectivity on new drug discovery. Pharmacol. Rev. *62*, 265–304. https://doi.org/10.1124/pr.108.000992.
16. De Smet, F., Christopoulos, A., and Carmeliet, P. (2014). Allosteric targeting of receptor tyrosine kinases. Nat. Biotechnol. *32*, 1113–1120. https://doi.org/10.1038/nbt.3028.
17. Christopoulos, A., May, L.T., Avlani, V.A., and Sexton, P.M. (2004). G-protein-coupled receptor allosterism: the promise and the problem(s). Biochem. Soc. Trans. *32*, 873–877. https://doi.org/10.1042/BST0320873.
18. Wenthur, C.J., Gentry, P.R., Mathews, T.P., and Lindsley, C.W. (2014). Drugs for allosteric sites on receptors. Annu. Rev. Pharmacol. Toxicol. *54*, 165–184. https://doi.org/10.1146/annurev-pharmtox-010611-134525.
19. Fox, S., Farr-Jones, S., Sopchak, L., Boggs, A., Nicely, H.W., Khoury, R., and Biros, M. (2006). High-throughput screening: update on practices and success. J. Biomol. Screen. *11*, 864–869. https://doi.org/10.1177/1087057106292473.

20. Andricopulo, A.D., Salum, L.B., and Abraham, D.J. (2009). Structure-Based Drug Design Strategies in Medicinal Chemistry. https://doi.org/10.2174/156802609789207127.

21. Molek, P., Strukelj, B., and Bratkovic, T. (2011). Peptide phage display as a tool for drug discovery: targeting membrane receptors. Molecules (Basel, Switzerland) *16*, 857–887. https://doi.org/10.3390/molecules16010857.

22. Nussinov, R., and Tsai, C.-J. (2013). Allostery in disease and in drug discovery. Cell *153*, 293–305. https://doi.org/10.1016/j.cell.2013.03.034.

23. Hardy, J.A., and Wells, J.A. (2004). Searching for new allosteric sites in enzymes. Curr. Opin. Struct. Biol. *14*, 706–715. https://doi.org/10.1016/j.sbi.2004.10.009.

24. Erlanson, D.A., Wells, J.A., and Braisted, A.C. (2004). Tethering: fragment-based drug discovery. Annu. Rev. Biophys. Biomol. Struct. *33*, 199–223. https://doi.org/10.1146/annurev.biophys.33.110502.140409.

25. Selvaratnam, R., Chowdhury, S., VanSchouwen, B., and Melacini, G. (2011). Mapping allostery through the covariance analysis of NMR chemical shifts. Proc. Natl. Acad. Sci. U S A *108*, 6138. https://doi.org/10.1073/pnas.1017311108.

26. Oyen, D., Wechselberger, R., Srinivasan, V., Steyaert, J., and Barlow, J.N. (2013). Mechanistic analysis of allosteric and non-allosteric effects arising from nanobody binding to two epitopes of the dihydrofolate reductase of *Escherichia coli*. Biochim. Biophys. Acta *1834*, 2147–2157. https://doi.org/10.1016/j.bbapap.2013.07.010.

27. Rath, V.L., Ammirati, M., Danley, D.E., Ekstrom, J.L., Gibbs, E.M., Hynes, T.R., Mathiowetz, A.M., McPherson, R.K., Olson, T.V., Treadway, J.L., and Hoover, D.J. (2000). Human liver glycogen phosphorylase inhibitors bind at a new allosteric site. Chem. Biol. *7*, 677–682. https://doi.org/10.1016/S1074-5521(00)00004-1.

28. Wright, S.W., Carlo, A.A., Carty, M.D., Danley, D.E., Hageman, D.L., Karam, G.A., Levy, C.B., Mansour, M.N., Mathiowetz, A.M., Mc- Clure, L.D., et al. (2002). Anilinoquinazoline inhibitors of fructose 1,6-bisphosphatase bind at a novel allosteric site: synthesis, in vitro characterization, and x-ray crystallography. J. Med. Chem. *45*, 3865–3877. https://doi.org/10.1021/jm010496a.

29. Collier, G., and Ortiz, V. (2013). Emerging computational approaches for the study of protein allostery. Arch. Biochem. Biophys. *538*, 6–15. https://doi.org/10.1016/j.abb.2013.07.025.

30. Sheik Amamuddy, O., Veldman, W., Manyumwa, C., Khairallah, A., Agajanian, S., Oluyemi, O., Verkhivker, G., and Tastan Bishop, O. (2020). Integrated computational approaches and tools forallosteric drug discovery. Int. J. Mol. Sci. *21*, 847. https://doi.org/10.3390/ijms21030847.

31. Huang, Z., Zhu, L., Cao, Y., Wu, G., Liu, X., Chen, Y., Wang, Q., Shi, T., Zhao, Y., Wang, Y., et al. (2010). ASD: a comprehensive database of allosteric proteins and modulators. Nucleic Acids Res. *39*, D663–D669. https://doi.org/10.1093/nar/gkq1022.

32. Huang, Z., Mou, L., Shen, Q., Lu, S., Li, C., Liu, X., Wang, G., Li, S., Geng, L., Liu, Y., et al. (2013). ASD v2.0: updated content and novel features focusing on allosteric regulation. Nucleic Acids Res. *42*, D510–D516. https://doi.org/10.1093/nar/gkt1247.

33. Shen, Q., Wang, G., Li, S., Liu, X., Lu, S., Chen, Z., Song, K., Yan, J., Geng, L., Huang, Z., et al. (2015). ASD v3.0: unraveling allosteric regulation with structural mechanisms and biological networks. Nucleic Acids Res. *44*, D527–D535. https://doi.org/10.1093/nar/gkv902.

34. Huang, W., Wang, G., Shen, Q., Liu, X., Lu, S., Geng, L., Huang, Z., and Zhang, J. (2015). ASBench: benchmarking sets for allosteric discovery. Bioinformatics *31*, 2598–2600. https://doi.org/10.1093/bioinformatics/btv169.

35. Zlobin, A., Suplatov, D., Kopylov, K., and Švedas, V. (2019). CASBench: a benchmarking set of proteins with annotated catalytic and allosteric sites in their structures. Acta Naturae *11*, 74–80.

36. Daura, X. (2019). In Advances in the Computational Identification of Allosteric Sites and Pathways in Proteins BT - Protein Allostery in Drug Discovery, J. Zhang and R. Nussinov, eds. (Springer Singapore), pp. 141–169. https://doi.org/10.1007/978-981-13-8719-7_7.

37. Huang, W., Lu, S., Huang, Z., Liu, X., Mou, L., Luo, Y., Zhao, Y., Liu, Y., Chen, Z., Hou, T., and Zhang, J. (2013). Allosite: a method for predicting allosteric sites. Bioinformatics *29*, 2357–2359. https://doi.org/10.1093/bioinformatics/btt399.

38. Chen, A.S.-Y., Westwood, N.J., Brear, P., Rogers, G.W., Mavridis, L., and Mitchell, J.B.O. (2016). A random forest model for predicting allosteric and functional sites on proteins. Mol. Inform. *35*, 125–135. https://doi.org/10.1002/minf.201500108.

39. Fogha, J., Diharce, J., Obled, A., Aci-Séche, S., and Bonnet, P. (2020). Computational analysis of crystallization additives for the identification of new allosteric sites. ACS Omega *5*, 2114–2122. https://doi.org/10.1021/acsomega.9b02697.

40. Van Gunsteren, W.F., Bakowies, D., Baron, R., Chandrasekhar, I., Christen, M., Daura, X., Gee, P., Geerke, D.P., Glättli, A., Hünenberger, P.H., and Kastenholz, M.A. (2006). Biomolecular modeling: goals, problems, perspectives. Angew. Chem. Int. Ed. *45*, 4064–4092. https://doi.org/10.1002/anie.200502655.

41. Ghosh, A., and Vishveshwara, S. (2007). A study of communication pathways in methionyl- tRNA synthetase by molecular dynamics simulations and structure network analysis. Proc. Natl. Acad. Sci. U S A *104*, 15711–15716. https://doi.org/10.1073/pnas.0704459104.

42. Shukla, D., Meng, Y., Roux, B., and Pande, V.S. (2014). Activation pathway of Src kinase reveals intermediate states as targets for drug design. Nat. Commun. *5*, 3397. https://doi.org/10.1038/ncomms4397.

43. Hollingsworth, S.A., and Dror, R.O. (2018). Molecular dynamics simulation for all. Neuron *99*, 1129–1143. https://doi.org/10.1016/j.neuron.2018.08.011.

44. Atilgan, A.R., Durell, S.R., Jernigan, R.L., Demirel, M.C., Keskin, O., and Bahar, I. (2001). Anisotropy of fluctuation dynamics of proteins with an elastic network model. Biophys. J. *80*, 505–515. https://doi.org/10.1016/S0006-3495(01)76033-X.

45. Hinsen, K. (1998). Analysis of domain motions by approximate normal mode calculations. Proteins Struct. Funct. Bioinform. *33*, 417–429. https://doi.org/10.1002/(SICI)1097-0134(19981115)33:3<417::AID-PROT10>3.0.CO;2-8.

46. Doruker, P., Atilgan, A.R., and Bahar, I. (2000). Dynamics of proteins predicted by molecular dynamics simulations and analytical approaches: application to α-amylase inhibitor. Proteins Struct. Funct. Bioinform. *40*, 512–524. https://doi.org/10.1002/1097-0134(20000815)40:3<512::AID-PROT180>3.0.CO;2-M.

47. Khairallah, A., Ross, C.J., and Bishop, Ö.T. (2021). GTP cyclohydrolase I as a potential drug target: new insights into its allosteric modulation via normal mode analysis. J. Chem. Inf. Model. https://doi.org/10.1021/acs.jcim.1c00898.

48. Panjkovich, A., and Daura, X. (2012). Exploiting protein flexibility to predict the location of allosteric sites. BMC Bioinform. *13*, 273. https://doi.org/10.1186/1471-2105-13-273.

49. Panjkovich, A., and Daura, X. (2014). PARS: a web server for the prediction of protein allosteric and regulatory sites. Bioinformatics *30*, 1314–1315. https://doi.org/10.1093/bioinformatics/btu002.

50. Greener, J.G., and Sternberg, M.J.E. (2015). AlloPred: prediction of allosteric pockets on proteins using normal mode perturbation analysis. BMC Bioinform. *16*, 335. https://doi.org/10.1186/s12859-015-0771-1.

51. Song, K., Liu, X., Huang, W., Lu, S., Shen, Q., Zhang, L., and Zhang, J. (2017). Improved method for the identification and validation of allosteric sites. J. Chem. Inf. Model. *57*, 2358–2363. https://doi.org/10.1021/acs.jcim.7b00014.

52. Guarnera, E., and Berezovsky, I.N. (2016). Structure-based statistical mechanical model accounts for the causality and energetics of allosteric communication. PLoS Comput. Biol. *12*, e1004678. https://doi.org/10.1371/journal.pcbi.1004678.

53. Tee, W.-V., Guarnera, E., and Berezovsky, I.N. (2018). Reversing allosteric communication: from detecting allosteric sites to inducing and tuning targeted allosteric response. PLoS Comput. Biol. *14*, 1–26. https://doi.org/10.1371/journal.pcbi.1006228.

54. Guarnera, E., and Berezovsky, I.N. (2019a). Toward comprehensive allosteric control over protein activity. Structure *27*, 866–878.e1. https://doi.org/10.1016/j.str.2019.01.014.

55. Guarnera, E., and Berezovsky, I.N. (2019b). On the perturbation nature of allostery: sites, mutations, and signal modulation. Curr. Opin. Struct. Biol. *56*, 18–27. https://doi.org/10.1016/j.sbi.2018.10.008.

56. Bahar, I., Lezon, T.R., Yang, L.-W., and Eyal, E. (2010). Global dynamics of proteins: bridging between structure and function. Annu. Rev. Biophys. *39*, 23–42. https://doi.org/10.1146/annurev.biophys.093008.131258.

57. Amor, B.R., Schaub, M.T., Yaliraki, S.N., and Barahona, M. (2016). Prediction of allosteric sites and mediating interactions through bond-to-bond propensities. Nat. Commun. *7*, 1–13. https://doi.org/10.1038/ncomms12477.

58. Delmotte, A., Tate, E.W., Yaliraki, S.N., and Barahona, M. (2011). Protein multi-scale organization through graph partitioning and robustness analysis: application to the myosin–myosin light chain interaction. Phys. Biol. *8*, 55010. https://doi.org/10.1088/1478-3975/8/5/055010.

59. Amor, B., Yaliraki, S.N., Woscholski, R., and Barahona, M. (2014). Uncovering allosteric pathways in caspase-1 using Markov transient analysis and multiscale community detection. Mol. BioSystems *10*, 2247–2258. https://doi.org/10.1039/C4MB00088A.

60. Spielman, D.A., and Teng, S.-H. (2004). Nearly-linear time algorithms for graph partitioning, graph sparsification, and solving linear systems. In Proceedings of the Thirty-Sixth Annual ACM Symposium on Theory of Computing. STOC '04 (Association for Computing Machinery), pp. 81–90. https://doi.org/10.1145/1007352.1007372.

61. Kelner, J.A., Orecchia, L., Sidford, A., and Zhu, Z.A. (2013). A simple, combinatorial algorithm for solving SDD systems in nearly-linear time. In Proceedings of the Forty-Fifth Annual ACM Symposium on Theory of Computing. STOC '13 (Association for Computing Machinery), pp. 911–920. https://doi.org/10.1145/2488608.2488724.

62. Hodges, M., Barahona, M., and Yaliraki, S.N. (2018). Allostery and cooperativity in multimeric proteins: bond-to-bond propensities in ATCase. Sci. Rep. *8*, 1–14. https://doi.org/10.1038/s41598-018-27992-z.

63. Vianello, F. (2020). Computational Characterisation of Protein Interaction Sites: From Small Ligand Pockets to Large Domain Interfaces, Ph.D. thesis (Imperial College London). https://doi.org/10.25560/89838.

64. Strömich, L., Wu, N., Barahona, M., and Yaliraki, S.N. (2020). Allosteric hotspots in the main protease of SARS-CoV-2. bioRxiv, 2020. https://doi.org/10.1101/2020.11.06.369439.

65. Mersmann, S.F., Strömich, L., Song, F.J., Wu, N., Vianello, F., Barahona, M., and Yaliraki, S.N. (2021). ProteinLens: a web-based application for the analysis of allosteric signalling on atomistic graphs of biomolecules. Nucleic Acids Res. https://doi.org/10.1093/nar/gkab350.

66. Vitagliano, L., Adinolfi, S., Sica, F., Merlino, A., Zagari, A., and Mazzarella, L. (1999). A potential allosteric subsite generated by domain swapping in bovine seminal ribonuclease. J. Mol. Biol. *293*, 569–577. https://doi.org/10.1006/jmbi.1999.3158.

67. Dey, S., Hu, Z., Xu, X.L., Sacchettini, J.C., and Grant, G.A. (2007). The effect of hinge mutations on effector binding and domain rotation in *Escherichia coli* D-3-phosphoglycerate dehydrogenase. J. Biol. Chem. *282*, 18418–18426. https://doi.org/10.1074/JBC.M701174200.

68. Lukacs, C.M., Oikonomakos, N.G., Crowther, R.L., Hong, L.-N., Kammlott, R.U., Levin, W., Li, S., Liu, C.-M., Lucas-McGady, D., Pietranico, S., and Reik, L. (2006). The crystal structure of human muscle glycogen phosphorylase a with bound glucose and AMP: an intermediate conformation with T-state and R-state features. Proteins Struct. Funct. Bioinform. *63*, 1123–1126. https://doi.org/10.1002/prot.20939.

69. Oikonomakos, N.G., Schnier, J.B., Zographos, S.E., Skamnaki, V.T., Tsitsanou, K.E., and Johnson, L.N. (2000). Flavopiridol inhibits glycogen phosphorylase by binding at the inhibitor site. J. Biol. Chem. *275*, 34566–34573. https://doi.org/10.1074/jbc.M004485200.

70. Ciaccio, C., Coletta, A., De Sanctis, G., Marini, S., and Coletta, M. (2008). Cooperativity and allostery in haemoglobin function. IUBMB Life *60*, 112–123. https://doi.org/10.1002/iub.6.

71. Suplatov, D., and Švedas, V. (2015). Study of functional and allosteric sites in protein superfamilies. Acta Naturae 7, 34–45.

72. Sträter, N., Schnappauf, G., Braus, G., and Lipscomb, W.N. (1997). Mechanisms of catalysis and allosteric regulation of yeast chorismate mutase from crystal structures. Structure *5*, 1437–1452. https://doi.org/10.1016/S0969-2126(97)00294-3.

73. Liu, X., Lu, S., Song, K., Shen, Q., Ni, D., Li, Q., et al. (2020). Unraveling allosteric landscapes of allosterome with ASD. Nucleic Acids res. D394–D401. https://doi.org/10.1093/nar/gkz958.

74. Song, F., Barahona, M., and Sophia, Y.N. (2020). BagPype: A Python Package for the Construction of Atomistic, 491energy-Weighted Graphs from Biomolecular Structures, Manuscript in preparation.

75. Word, J., Lovell, S.C., Richardson, J.S., and Richardson, D.C. (1999). Asparagine and glutamine: using hydrogen atom contacts in the choice of side-chain amide orientation. J. Mol. Biol. *285*, 1735–1747. https://doi.org/10.1006/jmbi.1998.2401.

76. Huheey, J.E., Keiter, E.A., Keiter, R.L., and Medhi, O.K. (2006). Inorganic Chemistry: Principles of Structure and Reactivity (Pearson Education India).

77. Hunter, C.A., and Sanders, J.K.M. (1990). The nature of .pi.-.pi. interactions. J. Am. Chem. Soc. *112*, 5525–5534. https://doi.org/10.1021/ja00170a016.

78. Lin, M.S., Fawzi, N.L., and Head-Gordon, T. (2007). Hydrophobic potential of mean force as a solvation function for protein structure prediction. Structure *15*, 727–740. https://doi.org/10.1016/j.str.2007.05.004.

79. Mayo, S.L., Olafson, B.D., and Goddard, W.A., III (1990). DREIDING: a generic force field for molecular simulations. J. Phys. Chem. (USA) *94*, 26. https://doi.org/10.1021/j100389a010.

80. Jorgensen, W.L., and Tirado-Rives, J. (1988). The OPLS [optimized potentials for liquid simulations] potential functions for proteins, energy minimizations for crystals of cyclic peptides and crambin. J. Am. Chem. Soc. *110*, 1657–1666. https://doi.org/10.1021/ja00214a001.

81. Schaub, M.T., Lehmann, J., Yaliraki, S.N., and Barahona, M. (2014). Structure of complex networks: quantifying edge-to-edge relations by failure-induced flow redistribution. Netw. Sci. *2*, 66–89. https://doi.org/10.1017/nws.2014.4.

82. Biggs, N., Biggs, N.L., and Norman, B. (1993). Algebraic Graph Theory, 67 (Cambridge university press).

83. Lambiotte, R., Delvenne, J., and Barahona, M. (2014). Random walks, markov processes and the multiscale modular organization of complex networks. IEEE Trans. Netw. Sci. Eng. *1*, 76–90. https://doi.org/10.1109/TNSE.2015.2391998.

84. Koenker, R., and Hallock, K.F. (2001). Quantile regression. J. Econ. Perspect. *15*, 143–156. https://doi.org/10.1257/jep.15.4.143.

85. Efron, B., and Tibshirani, R. (1994). An Introduction to the Bootstrap, 1st Edition (Chapman and Hall/CRC).

Patterns, Volume *3*

Supplemental information

Prediction of allosteric sites and signaling: Insights from benchmarking datasets

Nan Wu, Léonie Strömich, and Sophia N. Yaliraki

Table S1: Performance of other computational methods in prediction of allosteric sites. These methods have used ASBench[1] and AlloSteric Database (ASD)[2] for method validation.

Methods	Prediction Accuracy	Remarks
PARS[3]	65%	The method was tested on 58 proteins collected from the ASD[2].
AlloPred[4]	59%	119 proteins were collected from ASBench[1] and relevant site information were retrieved from UniProt[5] and the Catalytic Site Atlas[6]. Only the chain(s) involving the orthosteric and allosteric sites and the connecting chain(s) were considered i.e. not the whole protein structure. An average prediction accuracy of 59% was achieved when tested on 40 proteins (79 was used for model training).
AllositePro[7]	51.7%	The 147 nonredundant allosteric sites from the Core-Diversity set of AS-Bench[1] were used in this study and 76 out of 147 allosteric sites was successfully predicted.
SBSMMA[8]	Not reported	41 proteins were selected based on the operational definition of allosteric sites in the paper from ASBench[1]. Predictive power is quantifiled by the area under the ROC curves (AUCs) and 28 out of 48 have an AUC above 0.6.

Table S5: Protein structure used from the CASBench database. Orthosteric and allosteric ligands and site residues can be found directly in the CASBench database.

cas number	PDB
**cas0001**	1nxe, 1nxg, 1owc, 4g6b, 4jad, 4jae, 4jaf, 4jag
**cas0002**	3i1y, 3i28, 3koo, 3otq, 5ahx, 5ai4, 5ai5, 5aia, 5ak4, 5ak5, 5ake, 5akh, 5akx, 5aky, 5ald, 5alf, 5alh, 5alm, 5aln, 5alo, 5alt, 5alu, 5alv, 5alw, 5aly, 5am0, 5am4, 5am5
**cas0003**	3ion, 3iop, 3rwp, 4rqk, 4rqv, 4rrv, 4xx9
**cas0004**	4ey5, 5hf6, 5hf8, 5hf9, 5hfa
**cas0010**	1i2d
**cas0011**	2ewn
**cas0015**	2ym4, 2ym8, 4fst, 4fsy, 4ft3, 4ft7, 4fta, 4ftn, 4fto, 4ftr, 4ftu, 4gh2
**cas0016**	3csm, 4csm
**cas0021**	3l9h, 4zhi
**cas0024**	4ald
**cas0027**	1cza, 1dgk
**cas0028**	1lld, 1lth
**cas0029**	1ldn
**cas0030**	2him, 2p2d
**cas0039**	3ddn
**cas0040**	1psd, 1yba, 2p9c, 2p9e, 2p9g, 2pa3
**cas0047**	4r1r
**cas0050**	1xtu, 1xtv
**cas0051**	11ba, 11bg, 1bsr, 1n3z, 1r3m, 1r5c, 1tq9, 3bcm, 3djo, 3djp, 3djq, 3djv, 3djx, 4n4c
**cas0052**	2z60
**cas0054**	2jfx, 2jfy, 2jfz, 4b1f
**cas0056**	3hmi

**cas0060**	1boz, 1dhf, 1dlr, 1dls, 1drf, 1hfp, 1hfq, 1hfr, 1kms, 1mvs, 1mvt, 1ohj, 1ohk, 1pd8, 1pd9, 1s3u, 1s3v, 1s3w, 1u71, 1u72, 2c2s, 2c2t, 2dhf, 2w3a, 2w3m, 3f8y, 3f8z, 3fs6, 3ghc, 3ghw, 3gi2, 3gyf, 3l3r, 3n0h, 3ntz, 3nu0, 3nxr, 3nxt, 3nxv, 3nxx, 3nxy, 3nzd, 3oaf, 3s3v, 3s7a, 4ddr, 4g95, 4kd7, 4keb, 4m6k, 4m6l, 4qjc, 5hpb, 5hqy, 5hqz, 5hsr, 5hsu, 5ht4, 5ht5, 5hui, 5hvb, 5hve
**cas0061**	1dre, 1ra2, 1ra3, 1ra8, 1rb2, 1rb3, 1rc4, 1rd7, 1re7, 1rg7, 1rh3, 1rx4, 1rx5, 1rx6, 1rx7, 3dau, 4ej1, 4fhb, 4i13, 4i1n, 4kjj, 4kjl, 4p3r, 4qle, 4qlg, 4x5f, 4x5g, 4x5h, 4x5i, 4x5j, 5cc9, 5ccc, 7dfr
**cas0067**	2psq, 3ril
**cas0070**	1ibc, 1ice, 1rwm, 1rwn, 1rwo, 1rwv, 2h4w, 2h4y, 2h51, 2h54, 2hbq, 2hbr, 2hby, 2hbz, 3d6f, 3d6h, 3d6m
**cas0071**	1i4o
**cas0074**	1ibv, 1ibw, 1pya
**cas0079**	1bzc, 1bzj, 1c83, 1c84, 1c85, 1c86, 1c87, 1c88, 1ecv, 1g1g, 1g7g, 1gfy, 1kak, 1kav, 1l8g, 1nwe, 1ptt, 1ptu, 1ptv, 1q1m, 1xbo, 2azr, 2b07, 2bge, 2cm7, 2cma, 2h4g, 2h4k, 2hb1, 2nt7, 2nta, 2qbp, 2qbq, 2qbr, 2qbs, 2veu, 2vey, 2zn7, 4i8n
**cas0080**	1i00, 1juj, 3ob7, 5hs3, 5x5q, 5x67
**cas0085**	1fuo, 1fup, 1fuq, 1kq7
**cas0086**	1gos, 1oj9, 1oja, 1ojc, 1s2q, 1s2y, 1s3b, 1s3e, 2bk3, 2bk4, 2bk5, 2byb, 2c64, 2c65, 2c66, 2c67, 2c70, 2c72, 2c73, 2c75, 2c76, 2v5z, 2v60, 2v61, 2vrl, 2vrm, 2vz2, 2xcg, 2xfn, 2xfo, 2xfp, 2xfq, 2xfu, 3po7, 3zyx, 4a79, 4a7a, 4crt, 5mrl
**cas0091**	1fin, 2b54, 2cch, 4ez7

References

1. Huang, W., G. Wang, Q. Shen, X. Liu, S. Lu, L. Geng, Z. Huang, and J. Zhang (Mar. 2015). “AS-Bench: benchmarking sets for allosteric discovery”. *Bioinformatics* 31.15, pp. 2598–2600. DOI: 10.1093/bioinformatics/btv169.
2. Huang, Z., L. Zhu, Y. Cao, G. Wu, X. Liu, Y. Chen, Q. Wang, T. Shi, Y. Zhao, Y. Wang, W. Li, Y. Li, H. Chen, G. Chen, and J. Zhang (Nov. 2010). “ASD: a comprehensive database of allosteric proteins and modulators”. *Nucleic Acids Research* 39.suppl_1, pp. D663–D669. DOI: 10.1093/nar/gkq1022.
3. Panjkovich, A. and X. Daura (2012). “Exploiting protein flexibility to predict the location of allosteric sites”. *BMC Bioinformatics* 13.1, p. 273. DOI: 10.1186/1471-2105-13-273.
4. Greener, J. G. and M. J. E. Sternberg (2015). “AlloPred: prediction of allosteric pockets on proteins using normal mode perturbation analysis”. *BMC Bioinformatics* 16.1, p. 335. DOI: 10.1186/s12859-015-0771-1.
5. Consortium, T. U. (Jan. 2015). “UniProt: a hub for protein information”. *Nucleic Acids Research* 43.D1, pp. D204–D212. DOI: 10.1093/nar/gku989.
6. Furnham, N., G. L. Holliday, T. A. P. de Beer, J. O. B. Jacobsen, W. R. Pearson, and J. M. Thornton (Jan. 2014). “The Catalytic Site Atlas 2.0: cataloging catalytic sites and residues identified in enzymes”. *Nucleic Acids Research* 42.D1, pp. D485–D489. DOI: 10.1093/nar/gkt1243.
7. Song, K., X. Liu, W. Huang, S. Lu, Q. Shen, L. Zhang, and J. Zhang (Sept. 2017). “Improved Method for the Identification and Validation of Allosteric Sites”. *Journal of Chemical Information and Modeling* 57.9, pp. 2358–2363. DOI: 10.1021/acs.jcim.7b00014.
8. Tee, W.-V., E. Guarnera, and I. N. Berezovsky (June 2018). “Reversing allosteric communication: From detecting allosteric sites to inducing and tuning targeted allosteric response”. *PLOS Computational Biology* 14.6, e1006228. DOI: 10.1371/journal.pcbi.1006228.
